# Intravascular laser irradiation of blood as novel migraine treatment: an observational study

**DOI:** 10.1186/s40001-023-01438-3

**Published:** 2023-10-25

**Authors:** Hsin-Hung Chen, Chun-Yu Lin, Shean-Jen Chen, Wan-Yun Huang, Chien-Wei Kuo, Shin-Tsu Chang

**Affiliations:** 1 Department of Medical Education and Research, Kaohsiung Veterans General Hospital, Kaohsiung, 813414 Taiwan; 2 College of Photonics, National Yang Ming Chiao Tung University, Tainan, 71150 Taiwan; 3 Taiwan Instrument Research Institute, National Applied Research Laboratories, Hsinchu, 300092 Taiwan; 4 Department of Physical Medicine and Rehabilitation, Kaohsiung Veterans General Hospital, Zuoying Dist, # 386, Dazhong 1St Rd, Kaohsiung, 813414 Taiwan; 5 Institute of Allied Health Sciences, College of Medicine, National Cheng Kung University, Tainan, 70119 Taiwan; 6 Department of Nuclear Medicine, Pingtung Veterans General Hospital, Pingtung 900, Taiwan; 7 Department of Physical Medicine and Rehabilitation, Tri-Service General Hospital, School of Medicine, National Defense Medical Center, Neihu District, # 161, Section 6, Minquan East Road, Taipei, 114201 Taiwan

**Keywords:** Migraine, Rehabilitation, ILIB, SPECT, Cerebral blood flow

## Abstract

**Background:**

Migraine is one of four major chronic diseases that cause disability. Decreases in regional cerebral blood flow (rCBF) occur during migraine attacks. Laser therapy is extensively employed in treating other vascular diseases; nevertheless, its effectiveness in migraine management remains largely unknown. Therefore, we evaluated the effect of low-level intravascular laser irradiation of blood (ILIB) therapy in patients with migraine.

**Methods:**

We performed an observational case–control study in 24 patients suffering from migraine. Patients were divided into an ILIB treatment group and a traditional rehabilitation group. This study performed clinical assessments and single-photon emission computed tomography (SPECT) prior to and after the treatment and 1 month later. Changes in rCBF-SPECT between groups and between timepoints were compared to clinical outcomes.

**Results:**

Nine patients undergoing rehabilitation and fifteen patients undergoing ILIB were studied from baseline to 1 month follow-up. The ILIB group, visual analog scale for pain (P = 0.001), Montreal Cognitive Assessment (P = 0.003), and Athens Insomnia Scale (P < 0.001) symptom scores significantly improved after treatment. SPECT imaging showed a 1.27 ± 0.27 fold increase in rCBF after ILIB treatment, and no significant differences in the rehabilitation group.

**Conclusions:**

Low-level ILIB therapy is associated with better clinical and vascular outcomes, and may be a feasible treatment option for migraine. Although our sample size was small, our data provide a starting point for migraine laser therapy research.

**Supplementary Information:**

The online version contains supplementary material available at 10.1186/s40001-023-01438-3.

## Background

Up to 15% of the worldwide adult population is affected by migraine, rendering this neurological disorder a prevalent affliction [1]. The Global Burden of Disease Study 2019 indicated that migraine ranked first in terms of the number of years young women live with the disorder and second in the number of years for which men and women of all ages live with the disorder [2]. Migraine is also demonstrated in the literature as a risk factor for ischemic stroke, commonly a cause of global morbidity and mortality [3].

While migraines, both with and without aura, were historically described as vascular headaches due to observed changes in cerebral blood flow [4], modern research posits them as complex disorders stemming from intertwined neural and vascular mechanisms. Initial neuronal disruptions, such as cortical spreading depression, can give rise to secondary vascular responses. The integral role of the trigeminovascular system in this context highlights the intricate dance between neural events and vascular changes in the migraine milieu [5, 6]. As indicated in the literature, the trigeminovascular system assumes an essential function in this complicated condition, which creates an opportunity for a new migraine therapy [7]. Single-photon emission computed tomography (SPECT) imaging is extensively executed to measure reductions in regional cerebral blood flow (rCBF) when migraine attacks occur [8, 9].

Despite the prevalence and severity of migraine, it is often neglected and undertreated.

Physical therapy options, such as laser therapy, are understudied in migraine research. Low-level laser therapy, or photobiomodulation, uses helium–neon (HeNe) laser to induce microvascular and macrovascular responses that facilitate tissue repair, alleviate pain, and stimulate acupuncture points [10]. A report in 1983 on low-power laser irradiation identified a considerable increase in the excretion of 5-hydroxyindoleacetic acid (5-HIAA) in urine after therapy [11]. Research has also demonstrated migraineurs exhibit higher levels of plasma 5-HIAA relative to healthy volunteers and patients with tension headaches [12]. Low-level intravascular laser irradiation of blood (ILIB) within the last 30 years has exhibited efficacy in treating vascular, cardiac, and other systemic diseases. However, to our knowledge, ILIB has never been employed to treat migraine. Therefore, our study used SPECT to investigate whether rCBF and migraine can be improved through ILIB therapy.

## Methods

### Study participants

**Fig. 1:**
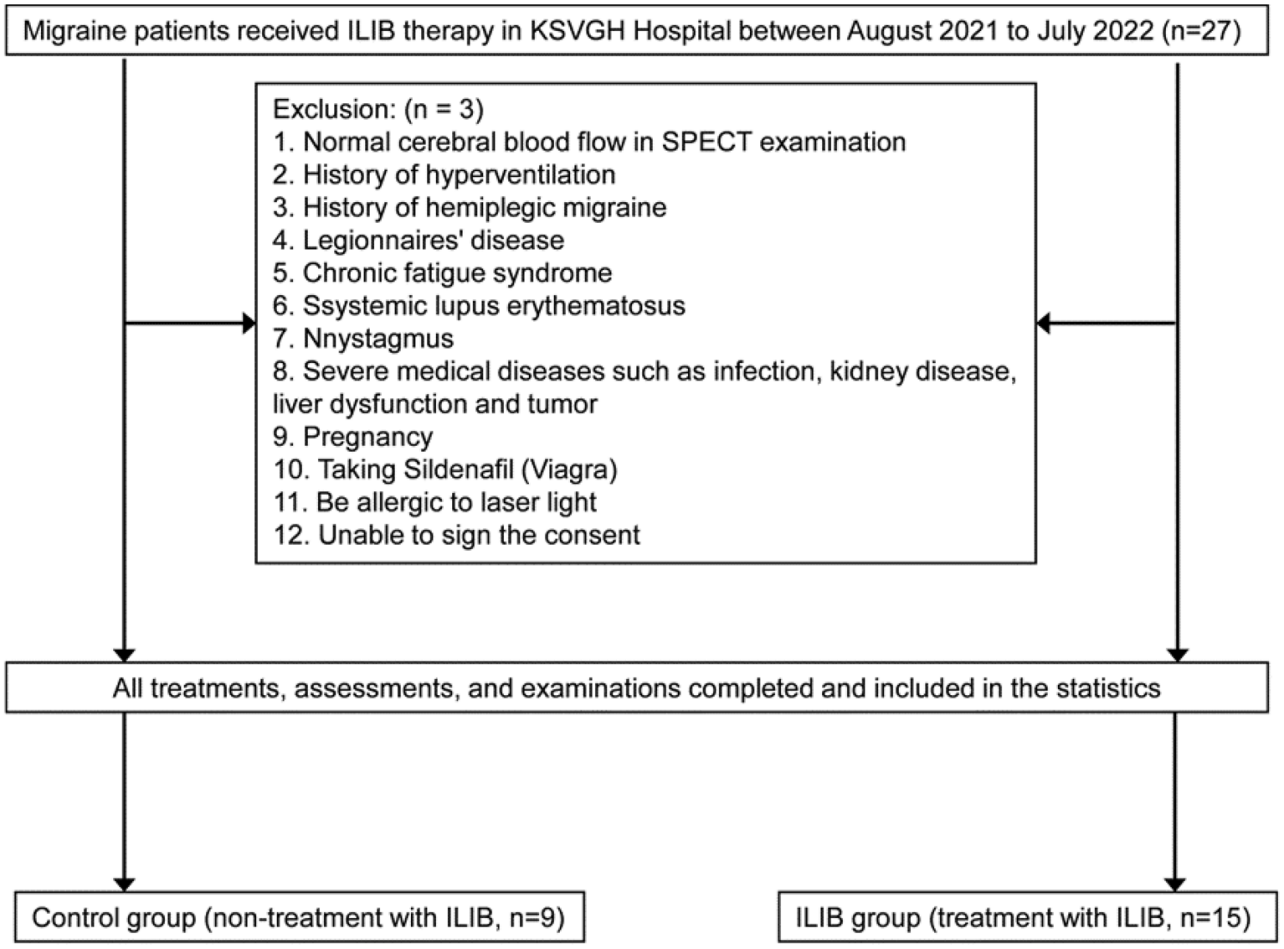
Study design flowchart. ILIB, intravascular laser irradiation of blood; KSVGH, Kaohsiung Veterans General Hospital; SPECT, single-photon emission computed tomography

Patients with migraine who were admitted to the Kaohsiung Veterans General Hospital’s Department of Physical Medicine and Rehabilitation from August 2021 to July 2022 were recruited for this study (Fig. 1). Patients were aged between 20 and 80 years; had a history of migraine and satisfied the *International Classification of Headache Disorders, Third Edition* criteria; had never received intravenous laser phototherapy; and had insufficient rCBF during a migraine attack, as determined through SPECT scanning. Those with a history of hyperventilation or hemiplegic migraine, Legionnaires’ disease, chronic fatigue syndrome, systemic lupus erythematosus, nystagmus, infection, or severe medical diseases (e.g., kidney disease, liver dysfunction, or cancer) were excluded. Additional exclusion criteria were the use of sildenafil, pregnancy, laser light allergy, and inability to provide consent.

### Study design

Patients were divided into two groups: traditional rehabilitation and ILIB treatment groups. Various clinical assessments were performed prior to treatment, immediately after treatment, and 1 month later for all patients. These tools applied for the aforementioned assessments included the visual analog scale (VAS) for pain, Montreal Cognitive Assessment (MoCA), Migraine Disability Assessment (MIDAS), and Athens Insomnia Scale (AIS) [13]. In addition, patients underwent SPECT imaging to determine rCBF at baseline and at a 1 month follow-up.

#### ILIB

Laser therapy was performed by our medical team. A cannula was inserted into a suitable vein (median antebrachial, accessory cephalic, or cephalic vein), followed by inserting a fiber into the cannula to provide a path for the laser. ILIB was performed using a helium–neon (He–Ne) laser illuminator, with a 632.8 nm, 1–5 mW/cm^2^ (TAIEX He–Ne Laser, YJ-ILIB-5, Bio-ILIB Human Energy Ltd, Taiwan). Laser power intensity was 0.51–2.55 W/cm^2^, energy was 3.6–18 J, and energy density was 1836.73–9183.67 J/cm^2^. Power intensity was adjusted based on the patient’s clinical responses [14]. Specifically: (1) Initial Dosage Setting Based on BMI: The starting output dosage for the laser was determined by the patient's body mass index (BMI). We adopted a ratio where the BMI was divided by 10 to determine the starting dose. For instance, a patient with a BMI of 25 would have an initial dosage set at 2.5 mW. (2) Incremental Dosage Adjustment: After the initial setting, the shooting dosage was increased by 0.1 mW every day to monitor any potential clinical reactions, ensuring we did not surpass the patient's threshold for laser power intensity. (3) Sleep Responses as a Clinical Marker: The quality and patterns of a patient's sleep served as the primary indicators for these adjustments. If a patient-reported sleep disturbances, such as feeling restless throughout the night or excessive excitement hindering their ability to sleep, it signaled that the previous day’s dosage was their maximum bearable limit. (4) Dosage Reduction Upon Identifying Threshold: Upon noting these sleep disturbances, the dosage was reverted to the previous day’s value and was consistently maintained for the rest of the treatment sessions. We executed ten irradiation sessions across 10 days, each lasting one hour per day.

### Traditional rehabilitation protocol

The traditional rehabilitation protocol comprised the following components: (1) Physical Therapy: Sessions included soft tissue massage, stretching, and strengthening exercises tailored to the patient's specific needs and pain points. Each session lasted 45 min and was conducted under the supervision of a licensed physiotherapist. (2) Biofeedback and Relaxation Techniques: These were integrated into the program to aid patients in recognizing and managing their pain triggers. Sessions were conducted once a month, lasting for 30 min. (3) Education and Counseling: Patients attended bi-weekly sessions where they were educated on migraine triggers, coping strategies, and preventative measures. Counseling also addressed the emotional and psychological aspects of living with migraines. (4) Home Exercise Program: Patients were provided with a set of exercises to perform at home daily. These exercises aimed to maintain and enhance the benefits obtained from the in-hospital physical therapy sessions. (5) Follow-up and Monitoring: After the initial intensive rehabilitation phase, patients were scheduled for biweekly follow-up sessions for the subsequent month to monitor progress and address any concerns.

### rCBF-SPECT imaging

To examine functional cortical reorganization and perfusion, we executed SPECT to assess the technetium-99 m ethyl cysteinate dimer distribution. Blood perfusion at rest was assessed in the left and right cerebral hemispheres at baseline and at the 1 month follow-up. The patient was advised not to smoke or consume caffeine, alcohol, or psychotropic drugs, which can affect cerebral blood flow. Changes in rCBF over the two assessment time points were determined.

The instrument provided hybrid CT and SPECT images in the same field of view. Registration between SPECT and CT images was necessary to resolve distortion during measurement. We assumed similarity between rigid-body and brain tissue; pre- and post-treatment CT images were similar for the ILIB group. Hence, the transformation matrix only accounted for displacement and rotation between the pre- and post-ILIB assessments. The MATLAB Image Processing Toolbox was used for CT and SPECT image registration. The pixel size of the CT images was changed to match that of the SPECT images and ensured all images had a resolution of 2.2 mm. An intensity-based automatic image registration algorithm was then applied to the modified CT images to calculate a transformation matrix. The transformation matrix was applied to the SPECT images to align the pre- and post-treatment scans. The grayscale value of each pixel in the aligned SPECT images was converted to a pseudo-color image by using a color look-up table.

rCBF was measured using a three-dimensional stereotactic region-of-interest (ROI) template and regulated brain SPECT images for quantification. The ROIs corresponded to seven bilateral areas: the parietal lobe, frontal lobe, basal ganglia, thalamus, temporal lobe, occipital lobe, and cerebellum.

### Statistical analysis

We used SPSS (version 22.0; IBM, Chicago, IL) for statistical analyses. Descriptive statistics were used to analyze patient demographic data. Categorical data on gender, education level, and living conditions were described using frequency distributions, whereas continuous variables, namely height, weight, and body mass index, were described using means and standard deviations. A paired t-test was conducted to identify differences between the preintervention and postintervention assessments. The preintervention assessment results were used as the benchmark group, and the postintervention data were analyzed and compared with the benchmark data.

## Results

### Participant baseline characteristics

This study recruited 24 patients with migraine. The rehabilitation and ILIB treatment groups comprised 9 and 15 patients, respectively. A total of 25% of the patients were men, and 75% were women (median age: 54 years, range 45–65 years). Table 1 presents the baseline demographic data on the participants. Of these variables, only weight was significantly different between the treatment groups.

**Table 1:** Demographic characteristics of all patients with migraine

Variable	Total (n = 24)	Migraine without ILIB (n = 9)	Migraine with ILIB (n = 15)	p value
Age (years, median, IQR)	57 (45–65)	64 (51–75)	56 (40–58)	0.199
Gender				
Male (n, %)	6 (25%)	2 (22.22%)	4 (26.67%)	1.000
Female (n, %)	18 (75%)	7 (77.78%)	11 (73.33%)	
Height (cm, mean ± SD)	162.42 ± 6.76	160.67 ± 5.34	163.47 ± 7.47	0.355
Weight (kg, mean ± SD)	64.09 ± 9.05	59.36 ± 8.26	66.93 ± 8.52	0.049
BMI (mean ± SD)	24.29 ± 3.23	23.06 ± 3.63	25.03 ± 2.83	0.084
Education level				
Elementary school (n, %)	1 (4.17%)	1 (11.11%)	0 (0%)	
Junior high school (n, %)	2 (8.33%)	2 (22.22%)	0 (0%)	
Senior high school (n, %)	3 (12.5%)	2 (22.22%)	1 (6.67%)	
Vocational high school (n, %)	5 (20.83%)	0 (0%)	5 (33.33%)	
Five-year junior college program (n, %)	3 (12.5%)	1 (11.11%)	2 (13.33%)	0.675
Bachelor (n, %)	7 (29.17%)	3 (33.33%)	4 (26.67%)	
Master (n, %)	2 (8.33%)	0 (0%)	2 (13.33%)	
PhD (n, %)	1 (4.17%)	0 (0%)	1 (6.67%)	
Resident situation				
Living with family (n, %)	22 (91.67%)	8 (88.89%)	14 (93.33%)	1.000
Living alone (n, %)	2 (8.33%)	1 (11.11%)	1 (6.67%)	

Categorical variables are listed as follows: number (percentage); Continuous variables are listed as: mean ± SD

ILIB, intravascular laser irradiation of blood; IQR, interquartile range; SD, standard deviation; BMI, body mass index; PhD, Doctor of Philosophy

### Assessment of clinical characteristics

After 10 ILIB treatment sessions, migraineurs exhibited significantly improved symptoms in the VAS of pain (2.63 ± 1.74 vs. 5.07 ± 1.59, *P* = 0.001), MoCA (28.33 ± 1.18 vs. 27.13 ± 2.03, *P* = 0.003), and AIS (3.60 ± 2.85 vs. 8.06 ± 4.42, *P* < 0.001) assessments (Table 2). At 1 month follow-up, the VAS of pain (3.30 ± 2.32 vs. 5.07 ± 1.59, *P* = 0.021), the MOCA (28.60 ± 1.68 vs. 27.13 ± 2.03, *P* = 0.034), the MIDAS (10.13 ± 18.62 vs. 30.40 ± 33.29, *P* = 0.033) and the AIS (3.80 ± 4.16 vs. 8.06 ± 4.42, *P* = 0.003) symptom changes were statistically significant. Moreover, AIS score was significantly better (3.80 ± 4.16 vs. 6.33 ± 4.44, *P* = 0.031) in the ILIB treatment compared to the rehabilitation group at 1 month follow-up.

**Table 2:** Assessment of pain intensity, cognition, disability, and insomnia between rehabilitation and ILIB treatment groups

Variable	Pre-treatment		Post-treatment		1 month post-treatment	
	Rehab	ILIB	Rehab	ILIB	Rehab	ILIB
VAS for pain (mean ± SD)	4.89 (2.55)	5.07 (1.59)	3.33 (2.78)	2.63 (1.74)^b^	3.61 (2.69)	3.30 (2.32)^c^
MOCA (mean ± SD)	24.56 (5.34)	27.13 (2.03)	25.11 (5.09)	28.33 (1.18)^b^	25.67 (5.12)	28.60 (1.68)^c^
MIDAS (mean ± SD)	74.67 (74.17)	30.40 (33.29)	62.33 (56.04)	32.2 (50.82)	30.67 (31.85)	10.13 (18.62)^c^
AIS (mean ± SD)	8.67 (5.87)	8.06 (4.42)	6.78 (7.03)	3.60 (2.85)^b^	6.33 (4.44)	3.80 (4.16)^a,c^

ILIB, intravascular laser irradiation of blood; VAS, Visual Analog Scale; MOCA, Montreal Cognitive Assessment; MIDAS, Migraine Disability Assessment; AIS, Athens Insomnia Scale

^a^A p-value of < 0.05 was significant in the experiment group of 1 month post-ILIB compared to the control group of 1 month post-ILIB

^b^A p-value of < 0.05 was significant in the experiment group of Psot-ILIB compared to the experiment group of Pre-ILIB

^c^A p-value of < 0.05 was significant in the experiment group of 1 month psot-ILIB compared to the experiment group of Pre-ILIB

### Assessment of rCBF-SPECT imaging

**Fig. 2:**
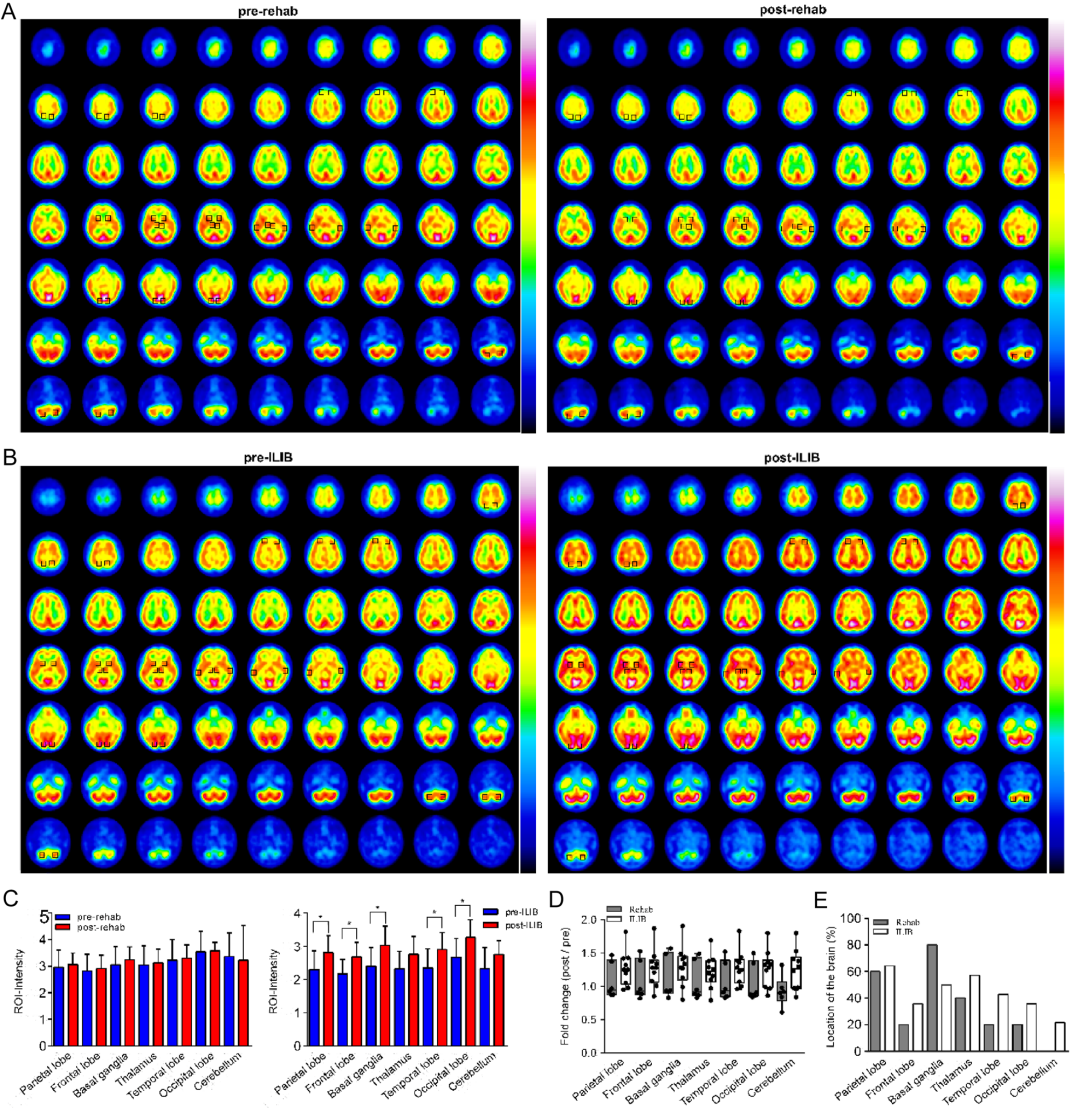
Regional cerebral blood flow imaging through single-photon emission computed tomography (rCBF-SPECT) scan of cerebral blood flow. **A** Rehabilitation group and **B** ILIB group. Preintervention and postintervention rCBF-SPECT did not differ significantly in the rehabilitation group, but rCBF was significantly higher in the patients with migraine after ILIB treatment. **C** Left panel presents the mean (error bars: standard deviation) rCBF differences between treatment groups for various brain regions. Right panel presents preintervention and postintervention rCBF differences in the ILIB group. **D** Between-group comparison of changes in rCBF in various brain regions. **E** Comparison of changes in region-specific rCBF between rehabilitation and ILIB groups. A significant difference was observed in rCBF before and after ILIB treatment. ILIB, intravascular laser irradiation of blood; Rehab, rehabilitation; ROI, region of interest.

ROI differences in rCBF-SPECT in rehabilitation (Fig. 2A) and ILIB (Fig. 2B) groups are shown in Fig. 2. We observed that the parietal lobe, frontal lobe, basal ganglia, temporal lobe, and occipital lobe were enhanced in rCBF-SPECT imaging after ILIB treatment (Fig. 2C). However, traditional rehabilitation before and after treatment did not change in rCBF-SPECT imaging. There were relative differences in changes from pretreatment to posttreatment status between rehab (Additional file 1: Video S1) and ILIB (Additional file 2: Video S2) groups. Ratio of posttreatment to pretreatment in rCBF-SPECT images had 1.27 ± 0.27 fold change by ILIB groups (Fig. 2D). Left and right rCBF were unevenly distributed during the migraine, but that improved after ILIB treatment in the location of the frontal lobe, thalamus, temporal lobe, occipital lobe, and cerebellum (Fig. 2E).

## Discussion

Our major findings showed that in patients with migraine, ILIB therapy improved pain, cognition, disability, insomnia, and rCBF. ILIB was first introduced as a therapy by Russian scientists in the 1970s [15] and remains a novel treatment method. It still represents a novel treatment modality. Currently, there are no established clinical ILIB strategies. However, since 2000, the efficacy of ILIB in various medical conditions, such as cardiovascular disease, dyslipidemia, type 2 diabetes, hepatic insufficiency syndrome, autoimmune diseases, and rheumatoid arthritis, has been observed, as reviewed by Tome et al. [16] ILIB engenders analgesic, sedative, and spasmolytic effects. It also affects all systems, causing widespread irradiation through circulation. Despite these benefits, ILIB has not been used to treat migraine. Our previous case report showed that ILIB could improve perfusion of the contralateral cerebellum during diaschisis after an ischemic stroke event [17], or traumatic brain injury [14], and improve sleep quality in patients with musculoskeletal pain [18, 19]. In the current study, we demonstrated ILIB-associated improvements in pain, cognition, disability, insomnia, and rCBF in patients with migraine.

For the assessment of migraine, we used four assessment scales. The pain assessment with VAS, cognitive assessment with MOCA, and insomnia assessment with AIS were improved after 10 sessions of ILIB treatment until up to 1 month, and future disability assessment with MIDAS ameliorated at 1 month posttreatment with ILIB. MOCA is a cognitive screening tool that accurately measures cognitive impairment in neurological disorders, including migraine. Santangelo et al. [20] indicated that the frequency with which migraine attacks occur or the duration of the disorder is not significantly correlated with cognitive performance, a finding that is consistent with the results of most studies [21, 22]. Conversely, Huang et al. observed an association between migraine and cognitive dysfunction, especially regarding headache duration and frequency [23]. Formerly, patients usually focused on the pain of the migraine and overlooked the impairment in cognitive function. However, our study revealed that ILIB treatment could improve both pain and cognitive dysfunction. In addition, the MIDAS assesses headache-related severity of disability to improve the degree of disability from migraine attacks [24]. Lipton et al. indicated that erenumab effectively reduces the number of monthly migraine attack days [25]. OnabotulinumtoxinA treatments improve disability symptoms, including headache pain frequency after 6 months [26] and 12 weeks [27]. Moreover, topiramate could significantly reduce monthly migraine attack days, and migraine-related disability by MIDAS scores significantly improved [28, 29]. Similarly, in our study, 10 sessions of ILIB treatment improved migraine-related disability for 1 month. Insomnia and other sleep disorders frequently occur together with migraine, and migraine disability and sleep quality have a bidirectional association. Insomnia, which is affected by headache and migraine attacks, reduces quality of life, can negatively affect the workforce, and creates an economic burden. We observed that ILIB treatment improves sleep quality in terms of AIS scores.

The typical migraine pathophysiology involves an abnormal stimulation of the trigeminovascular system in the meninges, causing neurogenic inflammation, which explains the anti-inflammatory effect of onabotulinumtoxin A during migraine attacks [30]. Vasoactive peptide (e.g., calcitonin gene-related peptide [CGRP]) release into extracerebral circulation has been observed in patients with migraine. Erenumab was the first CGRP monoclonal antibody available in the United Kingdom [31] and Germany [32]. However, Robblee et al. indicated that 28 (27.7%) of 101 patients discontinued erenumab, citing its ineffectiveness (39.3%, 11/28) or adverse effects (42.9%, 12/28) as reasons [33]. Although the migraine etiology is complex, hypoperfusion may trigger both migraines and strokes. This indicates that migraine and stroke may be a part of a spectrum of vascular complications in a group of patients with comorbid vascular conditions [34]. Imaging techniques such as SPECT [35] may result in the development of novel diagnostic and therapeutic interventions to improve quality of life for patients with migraine. This study demonstrated the usefulness of SPECT in assessing vascular changes. Although our data indicate that migraine severity improves with low-level laser therapy, additional changes in other areas, such as medication overuse, may lead to stronger effects on patients’ disability and quality of life.

Our study has several strengths and limitations. Firstly, our use of four different assessment scales for migraine and other factors provided thorough clinical classification. Unfortunately, we did not record specific information about migraine pain symptoms, such as the location and frequency. Further, this study only assessed patients for 1 month after treatment. Our small sample size did not allow us to further categorize patients with migraine; however, the fact that we identified statistically significant results across measures in a small sample is encouraging for future research. In summary, our prospective study identified the beneficial effects of ILIB therapy on rCBF and clinical symptoms in patients with migraine. Treatment that improves pain intensity, cognition, disability, insomnia, and neurovascular functioning warrants the pursuit of clinical trials in ILIB for migraine.

### Supplementary Information

**Additional file 1: Video S1.** Video S1 demonstrates that the cerebral blood flow changed from pre-treatment to post-treatment status in one participant of the rehabilitation group.

**Additional file 2: Video S2.** Video S2 demonstrates that the cerebral blood flow changed from pre-treatment to post-treatment status in one participant of the ILIB group.

## Data Availability

Written requests for access to the data reported in this paper will be considered by HHC and STC a decision made about the appropriateness of the use of data. If the use is appropriate, a data-sharing agreement will be put in place before a fully de-identified version of the dataset used for analysis with individual participant data is made available.

## Abbreviations

SPECT: Single-photon emission computed tomography
rCBF: Regional cerebral blood flow
5-HIAA: 5-Hydroxyindoleacetic acid
ILIB: Intravascular laser irradiation of blood
VAS: Visual analog scale
MoCA: Montreal cognitive assessment
MIDAS: Migraine disability assessment
AIS: Athens Insomnia Scale
ROI: Region-of-interest
CGRP: Calcitonin gene-related peptide
